# An Experimental 10-Port Microwave System for Brain Stroke Diagnosis—Potentials and Limitations

**DOI:** 10.3390/s25144360

**Published:** 2025-07-12

**Authors:** Tomas Pokorny, Jan Redr, Hana Laierova, Barbora Smahelova, Jakub Kollar

**Affiliations:** Department of Biomedical Technology, Faculty of Biomedical Engineering, Czech Technical University in Prague, 160 00 Praha, Czech Republic

**Keywords:** brain stroke, microwave devices, TSVD Born approximation, SVM

## Abstract

Microwave imaging systems show potential as replacements for commonly used stroke diagnostic systems. We developed and tested a 10-port microwave system on a liquid head phantom with ischemic and hemorrhagic strokes of varying sizes and positions. This system allows for visualization of changes in dielectric parameters using the TSVD Born approximation, enabling recognition of stroke position and size from the resulting images. The SVM algorithm effectively distinguishes between ischemic and hemorrhagic strokes, achieving 98% accuracy on experimental data, with 99% accuracy in ischemic scenarios and 97% in hemorrhagic scenarios. Using the TSVD Born algorithm, it was possible to precisely image changes in the absolute permittivity of different stroke locations; however, changes in stroke size were more apparent in the variations of absolute permittivity than in the reconstructed stroke size within the antenna plane. Outside this plane, changes in the S-parameters decreased depending on the distance and size of the stroke, making detection and classification more difficult. One ring of antennas around the head proved insufficient, prompting us to focus on developing a system with antennas positioned around the entire head.

## 1. Introduction

Stroke is an acute medical condition resulting from a disruption of blood flow to the brain. It affects up to 15 million people worldwide each year and is among the leading causes of both death (around 5 million annually) and long-term disability (another 5 million) [1]. There are two main types of stroke, each requiring significantly different treatments. Ischemic stroke is caused by the blockage of a cerebral blood vessel by a blood clot, whereas hemorrhagic stroke results from internal bleeding within the brain. Respiratory diseases increase the risk of stroke, and the COVID-19 pandemic has further contributed to its incidence. COVID-19 can lead to complications such as inflammation and endothelial damage, creating a prothrombotic state that promotes clot formation and increases the risk of stroke [2]. It is anticipated that the number of stroke cases will continue to rise [3]. Accurate and timely diagnosis is essential for initiating treatment as early as possible. Currently, strokes are typically diagnosed in hospitals using CT or MRI scans. However, transportation to the hospital takes valuable time—time during which faster intervention could significantly improve clinical outcomes [4].

Microwave imaging systems are emerging as a promising alternative to traditional CT and MRI scans for stroke diagnosis. These systems have the potential to be compact, portable, and cost-effective, making them suitable for use outside of hospital settings. Several research teams are actively working on their development. A notable example is the Strokefinder system developed by Medfield Diagnostics which is primarily designed to detect intracranial hemorrhages. In recent studies [5,6], the accuracy was reported to be over 90% in classifying and locating hemorrhagic strokes, although the accuracy for ischemic strokes was lower, at around 65%. A mobile scanner capable of reconstructing images and identifying stroke types is under development by EMTensor [7,8]. This device is relatively large and conducts continuous 24/7 diagnostics at the hospital bedside, making it impractical for ambulance use. EMVision has already launched two generations of microwave systems [9,10,11]. The first generation is intended for bedside monitoring of patients’ responses to therapy or surgical interventions, while the second generation is designed for pre-hospital diagnosis. Both systems rely on advanced imaging and classification algorithms, which are currently undergoing testing. Researchers in Italy [12,13,14] are also working on an experimental microwave system for real-time brain stroke monitoring. They are developing and testing algorithms for target imaging as well as algorithms for classifying stroke types and positions. Several other experimental systems have been presented [15,16,17,18,19,20]. However, their clinical implementation is still far from being realized.

Pre-hospital diagnostics, which could significantly improve clinical outcomes for stroke patients, are still unavailable. In ischemic stroke (iStroke), occurring in up to 85% of cases [1,21], approximately 2 million brain neurons perish every minute [22]. Early thrombolytic therapy of ischemic stroke is today an established procedure [23,24]. The initial 4.5 h following ischemic stroke onset constitute ‘the golden period’ for administering thrombolytic therapy to dissolve the clot [25,26]. In hemorrhagic stroke (hStroke), administration of thrombotic therapy raises the risk of hematoma expansion, which is associated with death and disability [27,28]. Hemorrhagic stroke is commonly associated with an acute increase in blood pressure, resulting in hematoma expansion [29]. Intensive blood pressure lowering and reversal of abnormal coagulation is feasible, safe, and significantly reduces hematoma expansion [30,31]. Rapid identification the type of stroke is crucial for early initiation of thrombotic therapy (in ischemic strokes) to dissolve the clot or to lower blood pressure and for early initiation of hemostatic therapy (in hemorrhagic strokes) to minimize hematoma expansion.

The objective of this study is to develop and evaluate a microwave imaging system for rapid pre-hospital stroke detection and classification of ischemic stroke types, enabling the safe initiation of thrombolytic therapy. This goal will be achieved by employing a machine learning algorithm (Support Vector Machine, SVM) and visualizing the stroke location based on changes in dielectric properties using the TSVD Born approximation method.

## 2. Materials and Methods

### 2.1. Microwave System for Head Stroke Measurements

**Figure 1:**
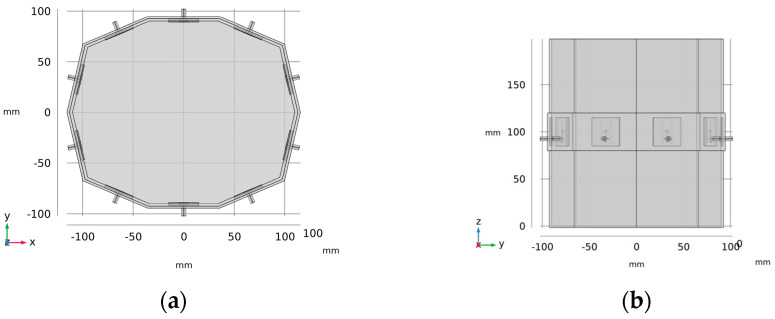
Numerical model of microwave system with 10 antennas in one plane: (**a**) top view and (**b**) side view.

**Figure 2:**
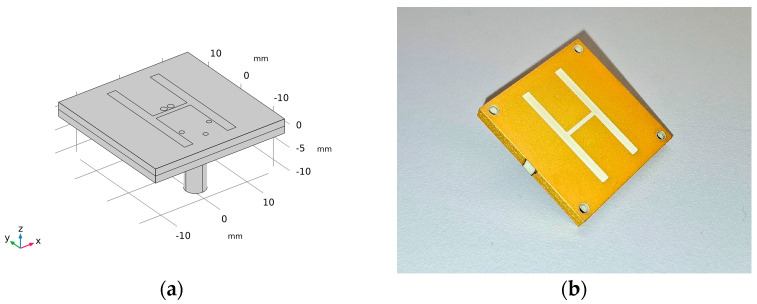
H-slot antenna developed for brain imaging purposes: (**a**) numerical model and (**b**) fabricated antenna.

The microwave system is designed as a 10-sided oval structure with an inner wall width of 66 mm, forming a container measuring 220 × 180 mm, with 3 mm thick walls and a height of 200 mm, as illustrated in Figure 1. Each wall houses a compact H-slot antenna placed at its center, specifically developed for microwave imaging of the head region (see Figure 2). The choice of slot antennas was based on a numerical comparison study [32] that evaluated three antenna types: bow-tie, slot, and rectangular waveguide-based antennas. The array consists of 10 antennas in total. The operating frequency was set to 1 GHz, as recommended by previous studies [33,34] and our previous studies [32,35].

The microwave system was fabricated using a Prusa i3 MK3s 3D printer (Prusa Research a.s., Prague, Czech Republic) and assembled with antennas using a two-component epoxy adhesive. To prevent water infiltration through potential microcracks in the 3D print, a thin epoxy layer was applied to the container. An equivalent numerical model was created in COMSOL Multiphysics 6.2. This model was used for numerical studies [35,36], as well as in the present study, to export electric fields for generating a linearized operator for the TSVD Born method.

### 2.2. Experimental Measurement

**Figure 3:**
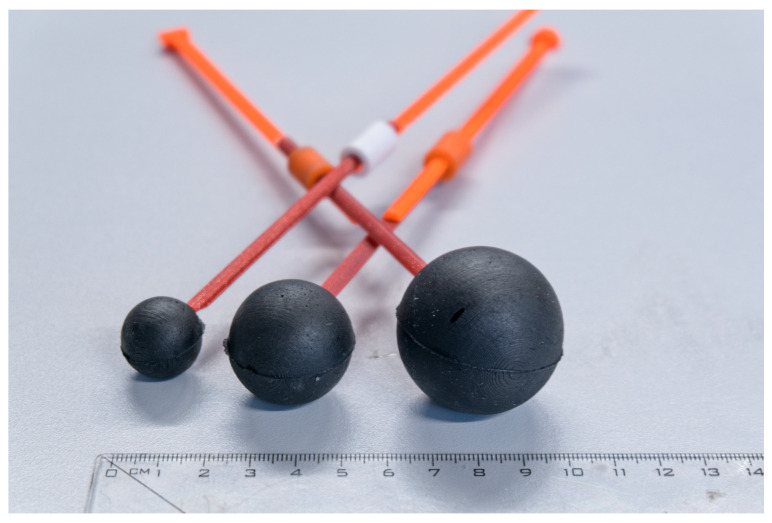
Stroke spherical phantoms of size 20, 30, and 40 mm in diameter.

The microwave system was filled with a homogeneous liquid phantom representing a human head, consisting of water, salt, and isopropanol [37]. The phantom was placed in a plastic container that also served as the antenna housing. Spherical stroke phantoms with diameters of 20 mm, 30 mm, and 40 mm, representing ischemic stroke (iStroke) and hemorrhagic stroke (hStroke), were created (see Figure 3). As described in [38], demountable molds were developed to produce these spherical stroke phantoms. These molds allowed for the accurate and reproducible manufacturing of the phantoms using mixtures of polyurethane rubber, graphite powder, carbon black powder, and acetone [37]. The composition ratios of these mixtures, expressed in weight percent, are listed Table 1. The dielectric parameters of the liquid human head phantom and stroke phantoms were measured using the SPEAG Dielectric Assessment Kit (DAK, Schmid & Partner Engineering AG, Zurich, Switzerland) with the DAK-3.5 probe in the frequency range of 0.5–3.0 GHz. The dielectric properties of each layer were compared with the target dielectric values obtained from the IT’IS material parameter database V3.0 [39] at a frequency of 1 GHz. The dielectric properties of the strokes and the head phantom are listed in Table 2.

**Figure 4:**
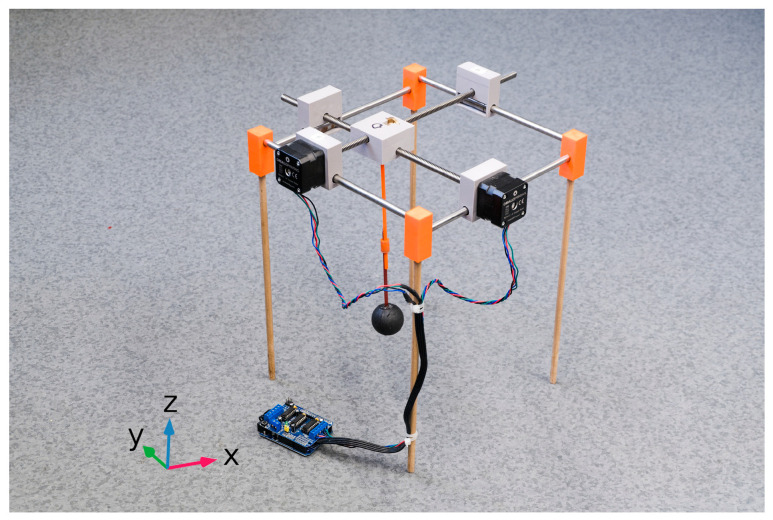
The computer-controlled stroke position system to change the position of the stroke phantom on the x and y axes.

The computer-controlled stroke positioning system was used to adjust the XY position of a stroke phantom on the head phantom (see Figure 4). The system consists of smoothed travel rods, stepper motors with trapezoidal rods, ball bearings, and linear bearings. Additional parts were 3D-printed on a Prusa i3 MK3 printer using PETG and bonded with polyurethane adhesive. Three-dimensionally printed stepper motor holders, with integrated linear bearings, provided stable mounting of the motors to the travel rods. The moving central part, also 3D-printed, includes space for two trapezoidal screws and incorporates a mechanism to hold the stroke phantom. Three-dimensionally printed corner components were used to fix the travel rods and support legs. Stepper motors were controlled via MATLAB 2023b scripts and the Arduino platform, enabling the system to adjust the position of the central component (stroke phantom) in 5 mm increments.

**Figure 5:**
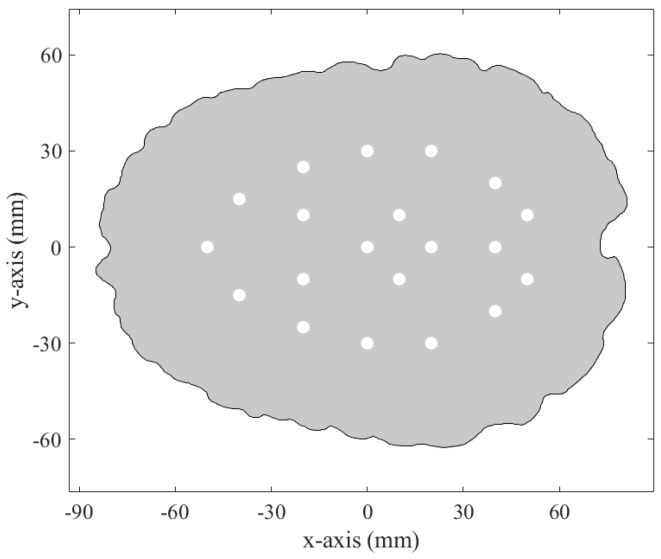
The 20 different x-y positions in the brain, indicated by white circles, where the stroke phantom was placed using the computer-controlled stroke positioning system. The z-axis position of the phantom was set to the center of the antennas.

Twenty different positions were selected along the xy-axis to position the spherical stroke phantom within the head phantom. Figure 5 shows the xy-coordinates of the stroke phantom at each of these locations. It is important to note that the z-axis of the stroke phantom is aligned with the plane in the center of the antennas.

Microwave measurements were performed using a custom MATLAB script to control the vector network analyzer (VNA) ZNB8 and the switching matrix ZN-Z84, both from Rohde & Schwarz (Munich, Germany). Prior to measurement, a full-port calibration was conducted using the automatic calibration unit ZN-Z153 (Rohde & Schwarz). Measurements were taken at a frequency of 1 GHz with an inter-frequency bandwidth of 10 Hz and an output power of 10 dBm. The measurement script initiated the movement of the stroke phantom to a predefined position and waited for the liquid level of the brain phantom to stabilize before starting the S-parameter measurements. Once the measurements were complete and the data saved, the stroke phantom was moved to the next predefined position, and the process was repeated. Finally, the stroke phantom was returned to its initial coordinates.

Two datasets were generated and are summarized in Table 3. The first training dataset consisted of 20 fixed stroke positions, 3 stroke sizes, and 40 noStroke measurements, resulting in a dataset size of 160 S-matrices. The second training dataset included 30 randomized stroke positions, again for 3 stroke sizes and 40 noStroke measurements, resulting in 220 S-matrices. The measurements were performed using a single homogeneous liquid phantom. To create the noStroke dataset, a noStroke measurement was performed each time a stroke phantom (either iStroke or hStroke) was exchanged. This approach was used to expand the dataset and include the noStroke scenario. It is important to note that although all noStroke measurements were acquired using the microwave system and the same homogeneous phantom without any stroke target, they still exhibited variability due to measurement noise. Including multiple noStroke measurements is therefore essential for training classification algorithms that can account for this natural variation.

### 2.3. Classification of Stroke Type Using SVM and PCA

For stroke detection and classification, a nonlinear Support Vector Machine (SVM) with a kernel function was used. The S-matrix contained only 55 independent elements, and it was represented by both real and imaginary parts, resulting in 110 observed features. Dimensionality reduction was performed using principal component analysis (PCA), which reduced the feature space to 20 principal components. These components were then used as input for SVM classification. A three-class classifier was developed to distinguish between three stroke categories: ischemic (iStroke), hemorrhagic (hStroke), and absence of stroke (noStroke). To enhance classification performance, cross-validation with k-fold = 5 was used during training, and an iterative hyperparameter optimization strategy available in MATLAB was applied to obtain the best classification results [35,36,38].

To evaluate the classification performance of the SVM algorithm, two independent datasets were used. The M1 dataset, containing 20 different stroke phantom positions evenly distributed within the brain phantom, was used exclusively for training. The M2 dataset, with stroke phantom positions randomly distributed throughout the brain, was used exclusively for testing. Classification performance was assessed by analyzing the confusion matrix and calculating Cohen’s kappa coefficient [35,36].

### 2.4. Differential Forward Scattering and Linear Inverse Scattering Solution

For imaging purposes, a differential microwave tomography algorithm presented in [40] was deployed. This algorithm utilizes the first-order Born approximation with truncated singular value decomposition (TSVD) to effectively regularize the inverse problem. The differential imaging problem is then expressed as follows:

(1) $${\Delta S}_{mn}{=L}_{e}\;\cdot \;\delta O,$$

where ${\Delta S}_{mn}$ is the differential S-matrix, ${\;L}_{e}$ is the linear operator, and δO is the object function.

The algorithm consists of two distinct stages: offline and online. During the offline stage, the linear operator is constructed from total electric fields of an empty system (Figure 1a) obtained from COMSOL Multiphysics simulation. This operator is subsequently decomposed into matrices *U*, *Σ*, and *V* via singular value decomposition (SVD). In the online stage, the differential S-matrix is generated by subtracting the measured S-matrix of an empty system from the S-matrix of a system containing a stroke phantom. The recovery of the change in the object function δO is achieved through a robust regularization method utilizing TSVD. In TSVD, the unknown object function is determined using the following inverse expression:

(2) $$\delta O=\sum_{n=1}^{nT}\frac{U^{\prime }\;\Delta S_{mn\;}}{\Sigma \;}V$$

where U, Σ, and V denote the matrices obtained from SVD of the linear operator and the upper index of the sum; nT represents the truncation index of TSVD [41].

In this study, the truncation index nT was set to 50, and the reconstructed images were generated based solely on the absolute value of the change in the object function δO [12]. Furthermore, a simple noise suppression technique was employed by applying a box blur convolution with a 3 × 3 kernel consisting of a matrix of ones (uniform averaging filter) to enhance the quality of the reconstructions.

## 3. Results

**Figure 6:**
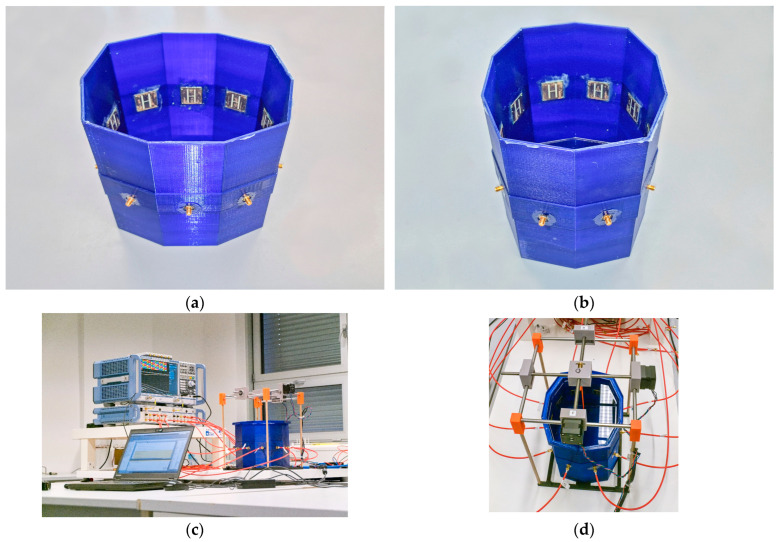
Microwave imaging system with 10 antennas in one plane: (**a**) top view and (**b**) side view. (**c**) Measuring setup with VNA, switching matrix, antenna array, and computer; (**d**) complete system in its operational state (including liquid head phantom, stroke mounted in position system, and antenna array).

We designed and built a microwave system with 10 antennas in one plane (see Figure 6). The system is designed for the purpose of stroke detection and classification.

Two datasets were used for testing stroke size and position detection using the TSVD Born algorithm, as well as for classifying ischemic and hemorrhagic strokes using the SVM algorithm. The datasets include measurements of different stroke types (iStroke and hStroke), as well as noStroke measurements. The number of S-matrix measurements (DataSet size) collected for each stroke type is summarized in Table 3.

**Figure 7:**
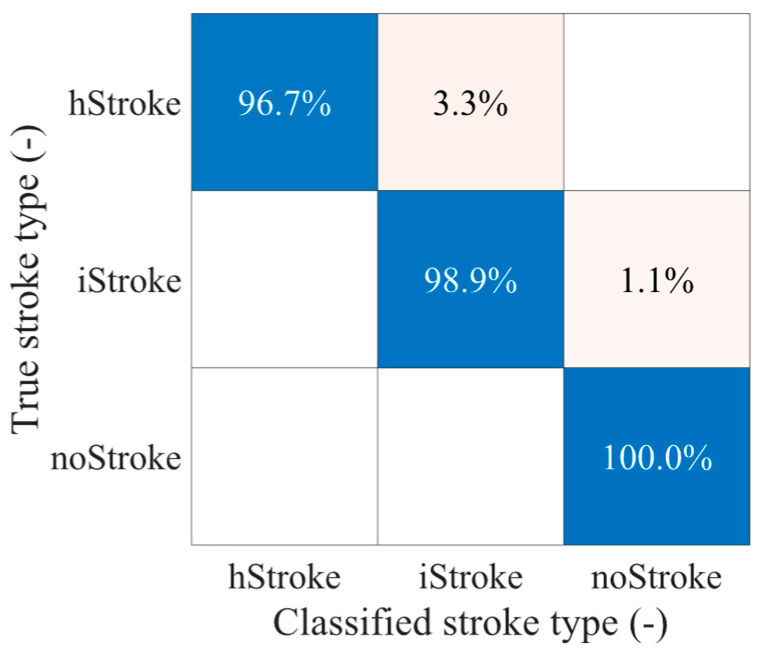
Confusion matrix for SVM classification for ischemic stroke, hemorrhagic stroke, and no-stroke scenario.

The SVM algorithm was tested on the M1 dataset, and the M2 dataset was used for classification. The overall classification accuracy reached 98.1%, corresponding to a Cohen’s kappa value of 0.95. This result was achieved after dimensionality reduction using PCA, reducing the feature space from 110 to 20 dimensions. Iterative hyperparameter optimization selected a Gaussian kernel with a kernel scale of 4.9 and a box constraint of 36.9. The confusion matrix for classification into individual classes is shown in Figure 7.

**Figure 8:**
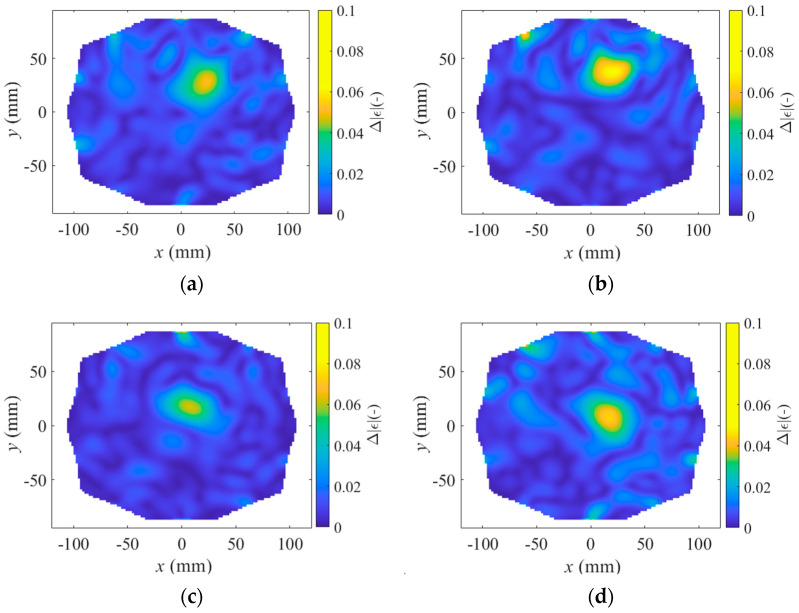
Result of TSVD Born imaging algorithm for (**a**,**c**) ischemic stroke and (**b**,**d**) hemorrhagic stroke of size 20 mm in diameter located (**a**,**b**) close to antennas at position (20, 30, 100) and located (**c**,**d**) deep in brain at position (10, 10, 100).

**Figure 9:**
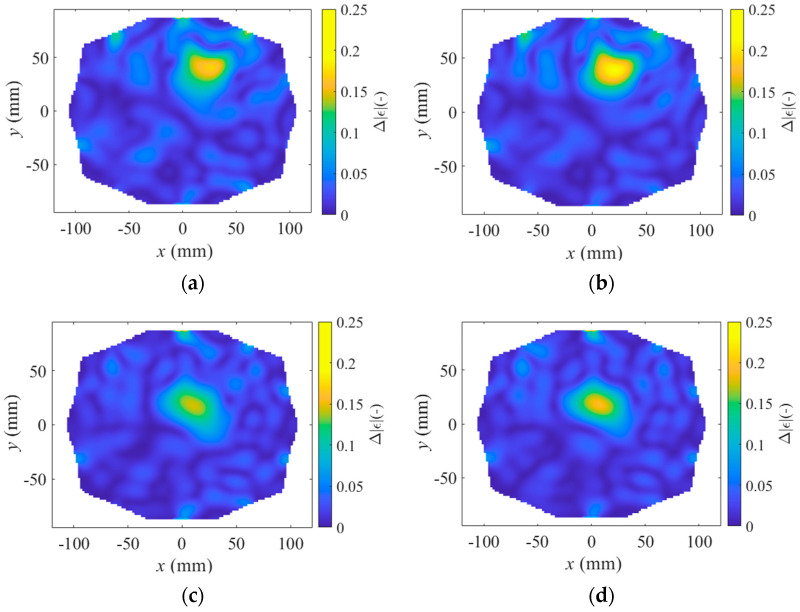
Result of TSVD Born imaging algorithm for (**a**,**c**) ischemic stroke and (**b**,**d**) hemorrhagic stroke of size 40 mm in diameter located (**a**,**b**) close to antennas at position (20, 30, 100) and located (**c**,**d**) deep in brain at position (10, 10, 100).

**Figure 10:**
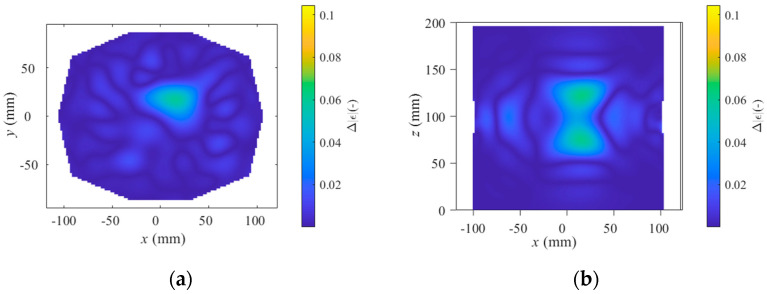
Result of TSVD Born algorithm for ischemic stroke of size 40 mm in diameter at position (20, 30, 70) located 30 mm under antenna plane. (**a**) XY view at stroke location (slice at Z = 70 mm), and (**b**) XZ view at corresponding Y-slice.

The TSVD Born algorithm was tested for stroke sizes of 20 mm and 40 mm in diameter. Imaging was compared for a stroke located superficially in the head, closer to the antennas, and a stroke located deeper in the head, further from the antennas. The results are shown in Figure 8, Figure 9 and Figure 10, presenting one case where the stroke is located outside the antenna plane.

## 4. Discussion

We developed a 10-port microwave system capable of detecting stroke types with 98% classification accuracy using a machine learning algorithm. The system also visualizes stroke positions using the TSVD Born approximation.

The presented microwave system has 10 antennas arranged in a single ring around the head. It operates within a limited frequency range, as the antennas are matched only in the 0.9 to 1.1 GHz range, which is suitable for microwave applications in the head region [32,33,34,35]. Measurements were performed using a vector analyzer and a switching matrix at a 1 GHz frequency. The results are based on the placement of the stroke at the level of the antennas, which represents a significant limitation of this study.

The stroke classification and imaging experiments were conducted using a homogeneous liquid head phantom, which served as a simplified model of the human head. The phantom consisted of a cylindrical plastic container—also used as the antenna housing—filled with a matching medium that mimicked the average dielectric properties of brain tissue. Stroke lesions were modeled as localized, homogeneous changes in dielectric properties, representing ischemic and hemorrhagic strokes. While polyurethane-based inclusions typically introduce higher measurement uncertainty compared to liquid phantoms, their greater dielectric contrast can potentially improve imaging and classification of stroke lesions. While the liquid brain phantom and rigid polyurethane strokes enabled controlled testing of the microwave system, both the head phantom and stroke model represent a substantial simplification, which is the main limitation of this study. In future work, to enable more comprehensive testing of the microwave system, we plan to use a multilayered brain phantom incorporating animal tissues to better represent the anatomical and dielectric properties of the brain. The time progression of stroke will be modeled as a gradual change in dielectric properties over time. Although physical phantoms are valuable for controlled experiments, they can never fully replicate the real anatomical complexity, physiological conditions, or tissue properties of the human brain. Therefore, final validation of the system will ultimately require clinical testing.

Stroke detection and classification were performed using an SVM algorithm with dimensionality reduction via PCA. The results demonstrate that by training the algorithm on only 20 stroke positions in the brain, it is possible to classify random stroke positions throughout the entire brain. The algorithm achieved a 98% classification success rate, correctly identifying hStroke 97% of the time and iStroke 99% of the time. Classification was performed on a homogeneous human head phantom with dielectric parameters matching those of a human head. Although classification may be more challenging with a more complex phantom, this study shows that a limited amount of training data are sufficient for accurate classification of random, unseen strokes.

This study also demonstrate that imaging changes in absolute permittivity using the TSVD Born algorithm can effectively detect the presence of a stroke. The results indicate that strokes located deeper in the head and closer to the center (coordinates 10, 10) cause a smaller change in absolute permittivity than those located farther from the center and closer to the antennas (coordinates 20, 30). Additionally, smaller strokes (20 mm in diameter, see Figure 8) result in a smaller change in absolute permittivity than larger strokes (40 mm in diameter, see Figure 9). Unfortunately, the change in stroke size is not clearly distinguishable in Figure 8 compared to Figure 9. The change in stroke size was primarily evident in the change in absolute permittivity values rather than in the diameter size of the stroke.

When the stroke is located outside the antenna plane, S-parameter variations decrease with distance and stroke size, and symmetric hourglass-shaped hotspots appear around the antenna axis (Figure 10), making it difficult to determine the stroke’s position along the Z-axis. In contrast, when the stroke is located within the antenna plane, no such hotspots appear along the Z-axis, and therefore only XY views are presented in Figure 8 and Figure 9. Although the stroke located outside the antenna plane was the same size (40 mm in diameter) as in Figure 9, the resulting changes in absolute permittivity are closer to those corresponding to the smaller stroke (20 mm in diameter) shown in Figure 8. The decrease in absolute permittivity contrast with increasing distance from the antenna plane causes the signal to fall below the noise level, making the SVM classification algorithm unable to reliably distinguish the stroke type.

The combination of SVM classification for identifying stroke type from measurements taken immediately after deploying the microwave system on a patient’s head, along with the visualization of changes in absolute permittivity using the TSVD Born algorithm over time, allows for both the diagnosis of stroke type and the monitoring of bleeding or perfusion progression after treatment initiation. As a result, the device is suitable for both pre-hospital diagnostics and bedside monitoring in hospital environments.

Microwave devices, due to their compact size and portability, have significant potential for rapid pre-hospital stroke diagnosis, enabling prompt treatment initiation and reducing the severity of the disease’s consequences. With non-ionizing radiation in the microwave spectrum, these devices also hold promise for continuous bedside monitoring of patients. If mass-produced, they could become affordable, extending their applicability to regions where advanced stroke care is not as widespread [42]. These devices could eventually become widespread—not only in hospitals and ambulances but also in settings like nursing homes or sports events. This broad availability would ensure rapid administration of thrombolytic therapy, intensive blood pressure management, and correction of abnormal coagulation, significantly reducing the time before stroke medications are administered.

In the FBME CTU team, we will continue developing the microwave system and algorithms. Our focus will be on creating a multi-port system with antennas positioned around the entire head. We aim to test SVM detection and classification of strokes located in all areas of the brain and design a comprehensive training dataset based on our research findings. Additionally, we plan to enhance the TSVD algorithm by incorporating 3D reconstruction of stroke size and location. Furthermore, we will explore methods for representing the distribution of dielectric parameters throughout the entire head to obtain an image encompassing all tissues.

## 5. Conclusions

We developed a 10-port system capable of accurately classifying stroke types with 98% accuracy using the SVM algorithm, along with visualizing stroke positions using the TSVD Born approximation algorithm. Although our system operates within a limited frequency range and is effective only with strokes located within the plane of the antennas, it shows promising potential for pre-hospital stroke diagnosis and continuous bedside stroke monitoring.

Our study has demonstrated the effectiveness of the SVM algorithm for stroke detection and classification, achieving high accuracy even with a small training dataset. Additionally, we have shown that the TSVD Born algorithm can successfully image absolute permittivity changes, allowing for the detection of stroke size and location.

Microwave devices have the potential to significantly improve the quality of care for stroke patients. Therefore, we will continue to develop more advanced systems and algorithms aimed at achieving even more accurate classifications and clearer imaging of strokes.

## Figures and Tables

**Table 1:** Phantom materials’ weight percentages.

Tissue Layer	Urethane Rubber (%)	Graphite Powder (%)	Carbon Black Powder (%)	Acetone (-)
Hemorrhagic stroke	61.5	22.0	3.5	13
Ischemic stroke	66.0	22.7	2.3	9
Liquid brain	61.15% deionized water + 38% isopropanol + 0.85% NaCl			

**Table 2:** The dielectric properties of the domain in the human head modes at 1 GHz. Measured data are supplemented with extended uncertainty (k = 2).

Tissue Layer	*ε_r_* (-)	*σ* (S/m)
Human head *	41.40 ± 0.71	1.04 ± 0.01
Ischemic stroke	31.72 ± 4.43	0.92 ± 0.07
Hemorrhagic stroke	52.73 ± 7.39	2.85 ± 0.58

* Average human head dielectric properties are given according to IEEE standard [24].

**Table 3:** Model parameters in datasets of experimental measurements.

DataSet (-)	Stroke Type (-)	Stroke Sizes (mm)	Stroke Positions (-)	Operating Frequency (GHz)	DataSet Size (-)
M1	hStroke	20, 30, 40	Fixed 20	1	60
	iStroke				60
	noStroke				40
M2	hStroke	20, 30, 40	Random 30 *	1	90
	iStroke				90
	noStroke				40

* Uniform probability density random number generator.

## Data Availability

The datasets of the measured S-parameters can be downloaded after filling out the following form: https://docs.google.com/forms/d/e/1FAIpQLSclU9oh8mqr3MF6LSmL6q9DPbFE5UL7wKeWBivZaXbxxpqrPw/viewform (accessed on 9 June 2025).
